# Comparison of Elastic Modulus Calculations in ASTM D7205 and CSA S806 for CFRP Rebar Under Elevated Temperature

**DOI:** 10.3390/polym17152143

**Published:** 2025-08-05

**Authors:** Seung-Beom Kang, Dae-Hee Kang, Wonchang Choi

**Affiliations:** Department of Architectural Engineering, Gachon University, Seongnam 13120, Republic of Korea; seungbum007@gachon.ac.kr (S.-B.K.); ilovejsdh@gachon.ac.kr (D.-H.K.)

**Keywords:** CFRP rebar, tensile strength, elastic modulus, elevated temperature

## Abstract

In this study, the elastic modulus of CFRP rebars under high-temperature conditions was evaluated in accordance with ASTM D7205 and CSA S806, and the differences between the two standards were compared and analyzed. CFRP rebars with diameters of 10 mm and 13 mm were tested, and tensile specimens were prepared following the procedures specified in both standards. Tensile tests were conducted at temperatures ranging from 25 °C to 650 °C using an electric furnace. Fracture morphology before and after testing, as well as microstructural changes, were examined through scanning electron microscopy (SEM). The results revealed that the ASTM standard determines the elastic modulus based on the initial linear portion of the stress–strain curve before the transition point, whereas the CSA standard includes the post-transition segment. At temperatures below 325 °C, the ASTM-derived modulus exhibited a lower coefficient of variation (COV) compared to the CSA-derived values, indicating a more stable performance. By applying the experimentally obtained modulus values to various prediction models, the model with the lowest average error was identified. These findings confirm that the elastic modulus of CFRP rebars can be reasonably predicted under elevated-temperature conditions using calibrated models.

## 1. Introduction

Fiber-reinforced polymer (FRP) reinforcement has gained attention as a potential alternative to steel reinforcement due to its excellent corrosion resistance and high tensile strength. Using FRP can improve durability and handling [1,2]. However, FRP reinforcement exhibits a noticeable reduction in both strength and stiffness when subjected to elevated temperatures, primarily due to the thermal degradation of the polymer matrix. This degradation poses a critical limitation in structural applications under high-temperature conditions or fire exposure scenarios [3]. Moreover, once the glass transition temperature (*T_g_*) is reached, the polymer matrix transitions from a glass to a rubbery state or begins to decompose, resulting in a significant reduction in the bond strength between the fibers and resin. This degradation of the fiber–matrix interface leads to a rapid decline in the overall mechanical performance of the FRP reinforcement [4].

Extensive research has investigated the tensile properties of FRP reinforcement under ambient and elevated temperatures [5]. The tensile strength and stiffness of FRP reinforcement decline sharply when exposed to elevated temperatures. The resulting critical temperature serves as a key parameter for evaluating the fire resistance performance of the material.

It has been reported that when the temperature exceeds the glass transition temperature (*T_g_*) of the resin, the matrix softens, resulting in a reduction in both the strength and stiffness of the composite [6]. Furthermore, once the resin decomposition temperature (*T_d_*) is surpassed, the mechanical performance of the FRP reinforcement deteriorates significantly. Additionally, previous studies have shown that larger-diameter FRP bars tend to exhibit higher critical temperatures due to delayed thermal penetration [7].

Carbon fiber-reinforced polymer (CFRP) reinforcement has demonstrated superior mechanical stability and bond performance at elevated temperatures when compared to glass fiber-reinforced polymer (GFRP) and basalt fiber-reinforced polymer (BFRP) [8]. In one study, CFRP rebars were exposed to high temperatures and subsequently cooled to room temperature under ambient conditions prior to tensile testing. The tensile strength recovered to approximately 83% of the value measured at high temperature, suggesting that the thermally softened resin underwent re-solidification during cooling, partially restoring its inter-fiber stress transfer capability [9]. The mechanical properties of FRP bars, including strength and elastic modulus, can vary depending on the type of polymeric matrix used [7,8].

In high-temperature tensile tests of GFRP reinforcement rods, the tensile strength was reduced by up to 96% relative to room temperature. However, the elastic modulus remained at approximately 66% of its room temperature value. This indicates that the elastic modulus is less sensitive to thermal degradation than tensile strength under elevated temperatures [10].

Furthermore, CFRP rods have been shown to maintain linear elastic behavior in the stress–strain response up to temperatures as high as 600 °C. Although the maximum strain decreased progressively with rising temperature, this behavior contrasts with that of conventional steel reinforcement, which typically exhibits increased ductility at high temperatures. The proposed relations for the tensile strength and elastic modulus of CFRP reinforcement have reportedly been useful indicators for evaluating the fire performance of FRP-reinforced concrete structures [11].

Tensile testing of CFRP tendons from room temperature up to 500 °C revealed a gradual reduction in tensile strength and elastic modulus with increasing temperature. The tensile strength decreased to about 50% of its room-temperature value at approximately 324 °C. This degradation in mechanical performance was attributed to the softening and decomposition of the resin as the temperature exceeded the glass transition temperature (*T_g_*) and the resin decomposition temperature (*T_d_*) [12]. Standard Test Method for the Tensile Properties of Fiber-Reinforced Polymer Matrix Composite Bars (ASTM D7205) and Design and Construction of Building Components with Fibre-Reinforced Polymers (CSA S806) are the most widely adopted standards for evaluating the tensile properties of FRP reinforcement. The two standards differ in their approaches to calculating the elastic modulus: ASTM utilizes a defined strain interval on the stress–strain curve, while CSA employs a stress-based interval. Although the materials and test conditions are identical, the modulus values and their variability can differ between the two standards. Differences in modulus values and variability may affect structural analysis results, depending on how material properties are assessed in design applications [13].

In Korea, the evaluation of tensile properties for FRP reinforcement is recommended based on the KS F ISO 10406-1 standard [14]. A recent study analyzing 13 mm diameter CFRP bars at 375 °C using the KS standard reported the emergence of a transition point within a specific temperature range on the stress–strain curve. This transition introduces sensitivity in the modulus calculation, making the results more susceptible to curve fluctuation and reducing reliability [15].

Furthermore, due to differences in the modulus calculation formula and correction method across each standard, comparative studies under controlled conditions have been suggested as essential.

Previous studies have quantified the mechanical behavior of FRP reinforcements at elevated temperatures. However, research remains limited regarding the applicability and reliability of tensile test standards and design criteria under thermal exposure [16]. Accordingly, this study tests larger-diameter CFRP rebars and compares the elastic modulus according to the ASTM D7205 and CSA S806 standards [17,18]. It quantitatively evaluates the differences between the guaranteed tensile strength and elastic modulus values, providing data to inform the design of FRP-reinforced structures under fire conditions.

The remainder of this paper is organized as follows. Section 2 describes the experimental program, including the test standards, CFRP rebar, and testing procedures. Section 3 presents and discusses the results, focusing on tensile strength, elastic modulus, and prediction models. Section 4 summarizes the main findings.

## 2. Experimental Program

### 2.1. ASTM and CSA Tensile Test Standard for FRP Rebar

To achieve this objective, CFRP reinforcement specimens with diameters of 10 mm and 13 mm were fabricated under identical conditions. Tensile tests were conducted at both room temperature and elevated temperatures to obtain tensile strength and elastic modulus values. The calculated modulus values were then compared between the two standards, and the variability of the results was analyzed to assess the applicability and reliability of ASTM D7205 [17] and CSA S806 [18] for evaluating the high-temperature mechanical performance of CFRP reinforcement.

ASTM D7205 [17], referenced in ACI 440.3R-12 [19], is a standardized test method used to determine the longitudinal tensile strength and elastic modulus of fiber-reinforced polymer (FRP) reinforcement employed in structural applications such as rebar, prestressing elements, and concrete components. According to the standard, FRP bars must be anchored at both ends of the specimen to prevent slippage during testing, and a minimum of five specimens per test condition is recommended.

The total specimen length is defined as the free length plus twice the anchor length. The standard recommends a free length of at least 40 times the nominal diameter of the specimen and a gauge length of at least 8 times the diameter. The testing speed should be selected such that failure occurs within 1 to 10 min after loading commences.

For elastic modulus determination, ASTM specifies a strain interval beginning at 0.001 and ending at 0.006. If the failure strain of the material is less than or equal to 0.012, the end point is modified to 50% of the measured ultimate strain. The ASTM standard presents the calculation formula for the elastic modulus and recommends that the coefficient of determination (r^2^) for the regression line be no less than 0.995, as determined by linear regression. ACI 440.1R-15 [20] further supplements the standard by providing explicit equations to calculate the guaranteed tensile strength and guaranteed elastic modulus for use in structural design. The formulas for guaranteed tensile strength and guaranteed elastic modulus are presented in Equations (1) and (2), respectively

(1) $$f_{fu}^{*}=f_{u,ave}-3\times SD$$

where f^*^_fu_ is the guaranteed tensile strength, *f_u,ave_* is the average tensile strength, and *SD* is the standard deviation.

(2) $$E_{f}=E_{f,ave}$$

where *E_f_* is the guaranteed elastic modulus, and *E_f,ave_* is the average value of the elastic modulus.

CSA S806 [18] is a Canadian standard for the design and construction of building components incorporating fiber-reinforced polymer (FRP) materials. It provides test methods for evaluating the mechanical properties of reinforcement bars, grids, sheets, laminates, and tendons. Similar to ASTM D7205 [17], the CSA standard requires cylindrical anchors to be installed at both ends of the specimen and recommends a minimum of five tests per test condition.

The total specimen length is defined as the free length plus twice the anchor length. The free length is recommended to be at least 40 times the nominal bar diameter, and the gauge length at least 5 times the diameter. The tensile loading rate is specified within the range of 250 to 500 MPa/min.

For the calculation of the elastic modulus, CSA S806 [18] specifies using the strain difference between the stress levels corresponding to 25% and 50% of the maximum tensile load. Additionally, CSA S807-10 [21] recommends the use of the coefficient of variation (COV) to determine the guaranteed tensile strength and guaranteed elastic modulus. The relevant equations are presented in Equations (3)–(5).

(3) $$f_{fu}^{*}=F_{t\_\mathrm{CSA}}\times f_{u,\mathrm{ave}}$$

where $F_{t\_CSA}=(1-1.645\times COV)/[1+(1.645\times COV/n^{1/2})]$; *COV* refers to the coefficient of variation, *n* is the number of specimens.

(4) $$(\mathrm{if}\;\mathrm{COV}\;>\;5\%)\;E_{f}=F_{E\_CSA}\times E_{f,ave}$$

where $F_{E\_CSA}=(1-1.645\times COV)/[1+(1.645\times COV/n^{1/2})]$

(5) $$(\mathrm{if}\;\mathrm{COV}\;<\;5\%)\;E_{f}=E_{f,ave}$$

### 2.2. CFRP Rebar

**Figure 1:**
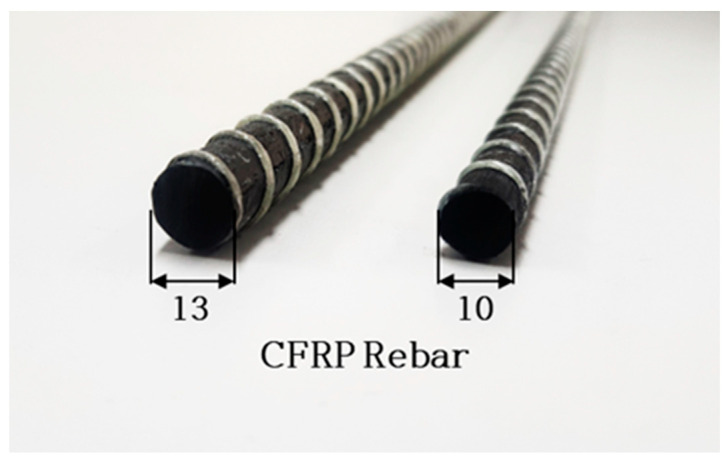
CFRP rebars used in this study.

The carbon fiber reinforcement used in this study was fabricated using a pultrusion process with H2550-24K carbon fiber from Company H (Seoul, Republic of Korea) and epoxy resin from Company K (Seoul, Republic of Korea). The outer surface of the reinforcement was wrapped with glass fiber, and the nominal diameters of the bars were 10 mm and 13 mm. The geometry of the reinforcement is illustrated in Figure 1.

To evaluate the thermal properties of the reinforcement, differential scanning calorimetry (DSC) tests were conducted to determine the glass transition temperature (*T_g_*) and the resin decomposition temperature (*T_d_*). The results are summarized in Table 1, along with the physical and mechanical properties of the reinforcement.

### 2.3. Test Specimen

Tensile test specimens were prepared in accordance with ASTM D7205 [17] and CSA S806 [18]. Both standards recommend that the free length of the specimen be at least 40 times the nominal diameter of the reinforcement. The anchor specifications, however, differ between the two standards. CSA S806 recommends the use of anchors with a minimum thickness of 5 mm, an inner diameter at least 10–14 mm larger than the reinforcement diameter, and a minimum length of 250 mm. In contrast, ASTM D7205 recommends using steel pipes with a minimum thickness of 2.87 mm and a length of 660 mm for specimens with 13 mm diameter reinforcement.

**Figure 2:**
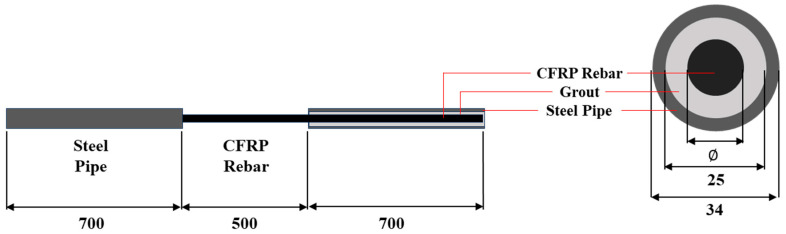
Details of the test specimen.

In this study, the free length was set to 500 mm. To prevent slippage at the gripping sections during testing, steel pipes with a length of 700 mm, an outer diameter of 34 mm, and a wall thickness of 4.5 mm were installed at both ends of the reinforcement. The interior of the steel pipes was filled with high-strength, non-shrink mortar from Sika, as illustrated in Figure 2.

### 2.4. Test Set Up

**Figure 3:**
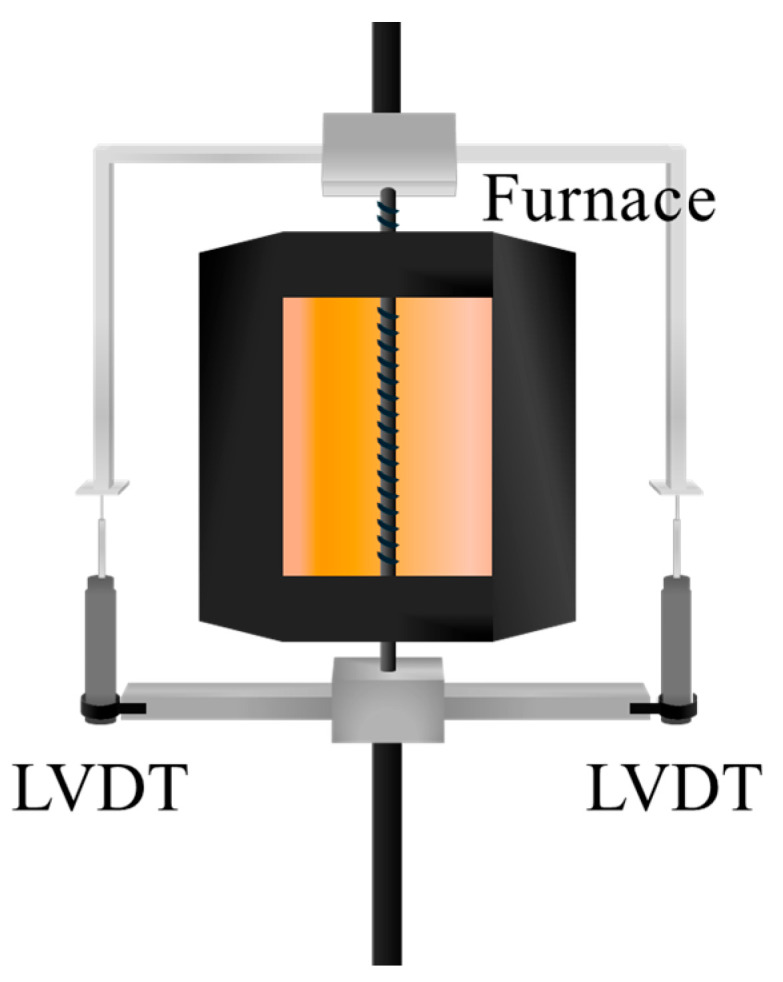
Test setup for CFRP rebar at elevated temperature.

**Figure 4:**
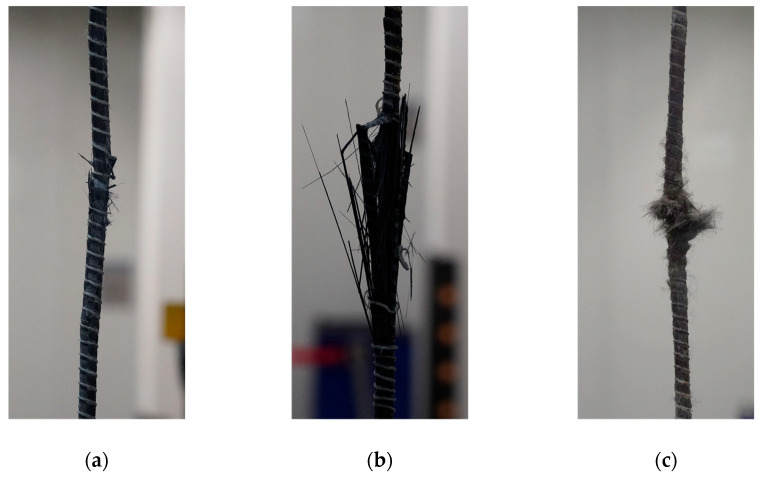
Failure mode of specimens after tensile test. (**a**) Type 1; (**b**) Type 2; (**c**) Type 3.

To create a high-temperature testing environment, a 4.2 kW electric furnace with a 200 mm heating zone was positioned at the center of the carbon fiber reinforcement. To ensure consistent thermal exposure, the furnace was preheated to the target temperature and then mounted at the center of the specimen. The target temperature was maintained for 30 min prior to testing [22], during which the furnace remained in place. Displacement was measured using two linear variable differential transformers (LVDTs), which were attached via jigs to both ends of the specimen’s free length. Tensile loading was applied in a displacement-controlled mode at a rate of 5 mm/min using a universal testing machine (UTM) with a capacity of 1200 kN. Photographs of the specimens before and after fracture under elevated temperatures are provided in Figure 3 and Figure 4, respectively. Figure 4a,b depict the fracture behavior of the fibers. In particular, Figure 4b shows that the outer glass fibers wrapping the rebar fractured first, followed by the failure of the internal fibers. This failure pattern was predominantly observed at temperatures below 300 °C. However, at temperatures exceeding the resin decomposition temperature of 300 °C, as shown in Figure 4c, the resin was thermally degraded or combusted, causing the fibers to become unraveled before fracturing.

The number of specimens tested at each temperature is summarized in Table 2. A total of five specimens were tested at temperatures ranging from 25 °C to 250 °C, and three specimens were tested at temperatures from 325 °C to 650 °C. The specimen identification code follows the format “D10–25–1,” where “D10” denotes the specimen diameter, “25 °C” indicates the test temperature, and “1” represents the specimen number.

## 3. Results and Discussion

### 3.1. Test Results

**Figure 5:**
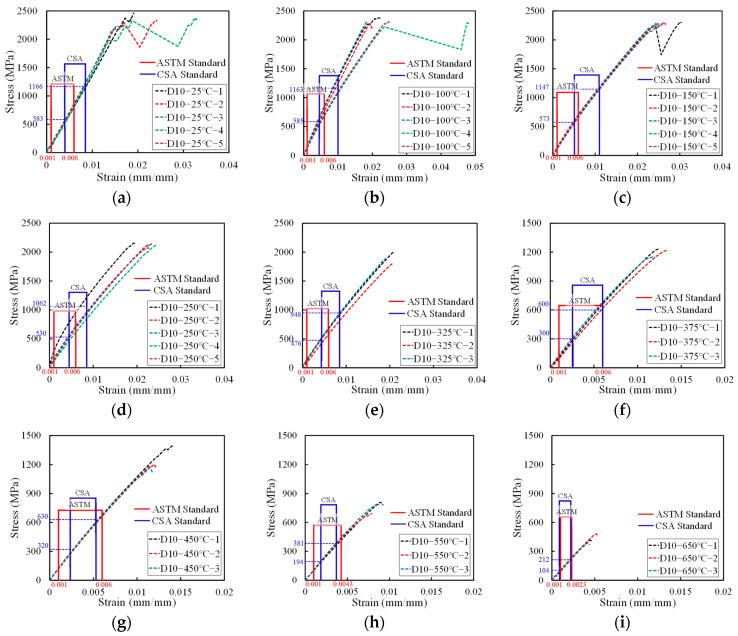
Stress−strain curves of D10 CFRP rebars at elevated temperatures. (**a**) D10−25 °C; (**b**) D10−100 °C; (**c**) D10−150 °C; (**d**) D10−250 °C; (**e**) D10−325 °C; (**f**) D10−375 °C; (**g**) D10−450 °C; (**h**) D10−550 °C; (**i**) D10−650 °C.

**Figure 6:**
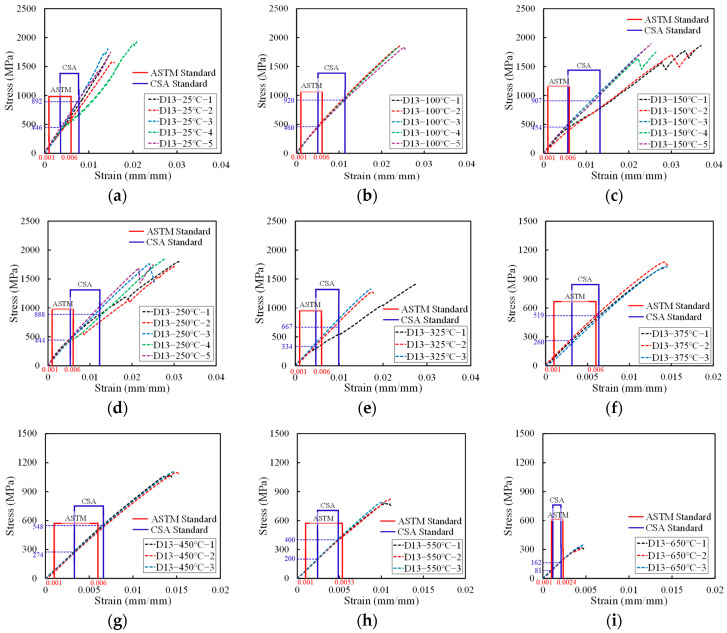
Stress−strain curves of D13 CFRP rebars at elevated temperatures. (**a**) D13−25 °C; (**b**) D13−100 °C; (**c**) D13−150 °C; (**d**) D13−250 °C; (**e**) D13−325 °C; (**f**) D13−375 °C; (**g**) D13−450 °C; (**h**) D13−550 °C; (**i**) D13−650 °C.

The results of the high-temperature tensile strength tests confirmed that all 70 specimens fractured within the 500 mm free length. The tensile strength data for the D10 CFRP bars, adopted from a previous study by Yun et al. [22], are presented in Table 3, while the results for the D13 CFRP bars tested in this study are shown in Table 4. The average elastic modulus values calculated for each bar type are summarized in Table 5 and Table 6. The intervals used for elastic modulus calculation are shown in Figure 5 and Figure 6.

Under the CSA standard, the elastic modulus is calculated based on the average stress corresponding to the strain between 25% and 50% of the maximum load. In contrast, the ASTM standard defines the modulus calculation interval based on strain values, specifically between 0.001 and 0.006, unless the ultimate strain is less than 0.012—in which case the upper limit is adjusted to 50% of the maximum strain.

For both D10 and D13 CFRP reinforcements, the ASTM-defined modulus intervals remained unchanged up to 450 °C, as the ultimate strain exceeded 0.012. However, at 550 °C and 650 °C, the ultimate strain was lower than 0.012, and accordingly, the modulus calculation interval was adjusted following ASTM guidelines. Since the CSA standard uses a load-based approach, whereas ASTM uses a strain-based approach, the calculated elastic modulus values differ between the two standards across different temperature conditions.

#### 3.1.1. Tensile Strength

Figure 5a–i and Figure 6a–i show the stress–strain curves for the tensile behavior of the D10 and D13 CFRP rebars at various temperatures from 25 °C to 650 °C. At 25 °C, the average tensile strength of the D10 carbon fiber reinforcement was measured to be 2330.8 MPa. The strength decreased by less than 9% up to 250 °C. Beyond 375 °C, a significant reduction in tensile strength was observed, with a decline of approximately 50%, and at 650 °C, the tensile strength had decreased by about 80% compared to room temperature. The ultimate strain increased with temperature up to 250 °C, after which it gradually declined from 325 °C onwards.

For the D13 carbon fiber reinforcement, the average tensile strength at room temperature was 1784.4 MPa. A slight increase in tensile strength was observed between 100 °C and 150 °C, which is attributed to the post-curing effect—thermal activation below the critical temperature that promotes further cross-linking within the resin matrix, thereby enhancing mechanical strength [23,24]. After 250 °C, the tensile strength began to decline, with a rapid decrease observed beyond 375 °C. At 650 °C, the tensile strength had decreased by more than 80% compared to its room temperature value. The ultimate strain increased markedly from room temperature to 150 °C, likely due to resin softening, and then decreased steadily from 250 °C.

When comparing the tensile strength of the D10 and D13 reinforcements across temperature conditions, the D13 bars exhibited approximately 23.4% lower tensile strength than the D10 bars at room temperature, with a similar trend persisting at elevated temperatures. This discrepancy is attributed to the shear lag effect, in which increased bar diameter leads to reduced stress transfer efficiency due to non-uniform stress distribution across the cross section [25]. When tensile force is applied through a steel grip, as shown in Figure 2, shear lag causes higher stress to develop in the outer fibers than in the inner ones. This non-uniform stress distribution can result in reduced tensile strength of FRP rebars [26].

The trend in ultimate strain—characterized by an initial increase followed by a gradual decline for both bar types—is interpreted because of the post-curing-induced resin hardening, which enhances inter-fiber bonding. However, upon exceeding the resin decomposition temperature (approximately 300 °C), the matrix begins to soften and degrade, thereby reducing its ability to effectively transfer stress among fibers.

#### 3.1.2. Elastic Modulus

The average value of the elastic modulus for the D13 carbon fiber reinforcement, calculated according to ASTM D7205, was 107.3 GPa at room temperature. Up to 325 °C, the modulus exhibited a decreasing trend, with a maximum reduction of approximately 27%. However, above 375 °C, the average modulus stabilized within the range of 82.1–84.9 GPa. Similarly, the elastic modulus determined in accordance with CSA S806 was 105.2 GPa at room temperature and decreased by up to 41% by 325 °C. Beyond 375 °C, the average modulus remained constant between 81.1 GPa and 84.9 GPa, exhibiting a trend consistent with that observed under the ASTM standard.

Between room temperature and 325 °C, both D10 and D13 CFRP reinforcements demonstrated that the ASTM standard defines the elastic modulus over an earlier segment of the stress–strain curve than the CSA standard. For the D10 reinforcement, the modulus values calculated using the ASTM procedure were between 0.1% and 9.8% higher than those derived from the CSA standard, with coefficients of variation (COV) within 5%, except at 100 °C and 250 °C. In the case of the D13 reinforcement, the difference between modulus values obtained by the two standards ranged from 0.3 GPa to 17.7 GPa at temperatures below 325 °C.

Moreover, the COV associated with the CSA-based modulus calculations ranged from 2.7% to 23.0%, showing an increasing trend with temperature. In contrast, the COVs derived from the ASTM-based modulus calculations remained consistently within 10%, indicating greater stability and less sensitivity to curve variation.

The observed increase in the coefficient of variation (COV) is attributed to the occurrence of a transition point in the stress–strain response near the glass transition temperature (Tg) and resin decomposition temperature (*T_d_*) of the carbon fiber reinforcement used in this study. This transition point means the strain point on the stress–strain curve where the slope changes. At temperatures below 325 °C, the discrepancy in the elastic modulus between the ASTM and CSA standards is likely due to differences in the modulus evaluation interval: the CSA standard captures data after the transition point, while the ASTM standard evaluates the linear portion preceding it.

The elevated COV and significant difference in modulus values obtained using the CSA method below 325 °C indicate that the CSA standard may have limitations in reliably applying its modulus calculation formula under certain high-temperature conditions. To address this issue, it is recommended that the CSA procedure incorporate a calibration method or revised modulus equation that accounts for material behavior in elevated-temperature environments.

Between 375 °C and 650 °C, the modulus evaluation interval defined by the CSA standard for both D10 and D13 specimens was found to be relatively shorter than that defined by the ASTM standard. However, in this temperature range, the difference in modulus values between the two standards remained within 1.4 GPa, and the COV was consistently below 3.3%, indicating a high level of agreement between the results.

**Figure 7:**
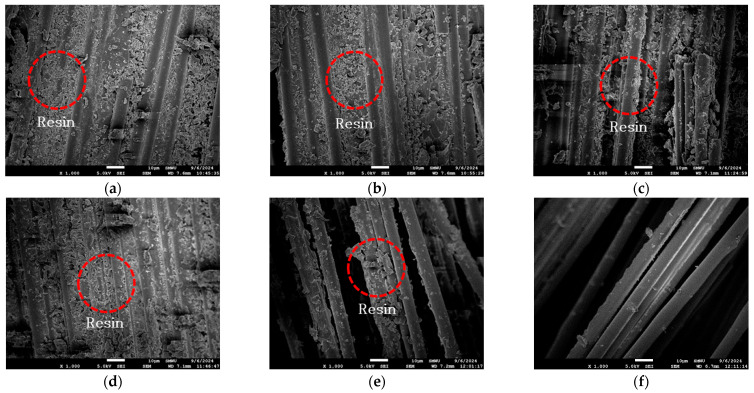

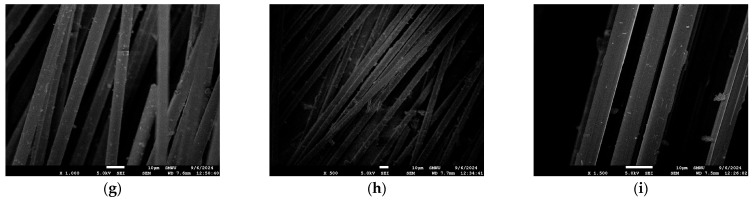
SEM images of CFRP rebars exposed to different elevated temperatures: (**a**) 25 °C; (**b**) 100 °C, (**c**) 150 °C; (**d**) 250 °C; (**e**) 325 °C; (**f**) 375 °C; (**g**) 450 °C; (**h**) 550 °C; (**i**) 650 °C. Red circles indicate resin.

As shown in Figure 7, scanning electron microscopy (SEM) images of the heated portion of the carbon fiber reinforcement after testing revealed that the resin remained at temperatures below 325 °C, whereas most of the resin was combusted at temperatures above 375 °C, leaving only the exposed fibers. Since resin plays a critical role in tensile performance by facilitating stress transfer between fibers, the presence of partially degraded resin at lower temperatures likely contributed to the higher COV and the emergence of transition points in the stress–strain curves.

The observed stabilization of the elastic modulus at temperatures above approximately 375 °C aligns with the findings of Zhang et al. (2021) [27], who reported that the tensile properties of fiber-reinforced composites are primarily governed by the fibers at elevated temperatures, with the influence of matrix softening and decomposition diminishing beyond the thermal degradation threshold [28]. In this study, it is interpreted that while the initial reduction in modulus is caused by resin softening, the subsequent stabilization is due to the dominance of carbon fibers in load-bearing behavior after matrix degradation.

#### 3.1.3. Guaranteed Tensile Strength and Guaranteed Elastic Modulus

According to ACI 440.1R-15, the guaranteed tensile strength and guaranteed elastic modulus are critical parameters in the design of concrete structures reinforced with FRP rebars. Therefore, in this study, the guaranteed tensile strength and guaranteed elastic modulus of CFRP rebars exposed to elevated temperatures were calculated. The differences between these guaranteed values and the average tensile strength and modulus were compared across various exposure temperatures, and a strength reduction factor was derived to support the design of FRP-reinforced concrete structures under high-temperature conditions. Table 7 presents the guaranteed tensile strength and elastic modulus values calculated in accordance with CSA S806 and ASTM D7205, while Table 8 summarizes the differences between the two standards.

### 3.2. Experimental Equation

The average elastic modulus calculated using the CSA standard was lower than that obtained using the ASTM standard and exhibited a higher coefficient of variation. Therefore, in this study, the elastic modulus prediction was based on the average values derived from the ASTM standard when evaluating high-temperature behavior. The purpose of employing an elastic modulus prediction equation is to address the limitation that experimental testing alone cannot capture all variations in the elastic modulus across a wide range of temperature conditions.

To evaluate the applicability of modulus prediction for D10 and D13 carbon fiber-reinforced bars, predictive equations from previous studies were applied, focusing on the ASTM standard as a reference. Specifically, the elastic modulus measured at room temperature was used to predict the elastic modulus at elevated temperatures for CFRP reinforcements.

Saafi (2002) proposed an empirical Equation (6) to account for the reduction in elastic modulus of CFRP reinforcement with increasing temperature [28].

(6) $$k_{E}=\left\{\begin{array}{c} 1\;\;\;\;\;\;\;\;\;\;\;\;\;\;\;\;\;\;\;\;\;\;\;\;\;\;\;\;\;\;\;\;\;\;\;\;\;\;\;\;\;\;\;\;\;\;\;\;\;\;\;\;\;\;\;\;\;\;\;\;\;\;\;(0\leq T\leq 100\;{}^{\circ}C)\\ 1.175-0.00175T\;\;\;\;\;\;\;\;\;\;\;\;\;\;\;\;\;\;\;\;\;\;\;\;\;\;\;(100\;{}^{\circ}C\leq T\leq 300\;{}^{\circ}C)\\ 1.625-0.00325T\;\;\;\;\;\;\;\;\;\;\;\;\;\;\;\;\;\;\;\;\;\;\;\;\;\;\;(300\;{}^{\circ}C\leq T\leq 500\;{}^{\circ}C)\\ 0\;\;\;\;\;\;\;\;\;\;\;\;\;\;\;\;\;\;\;\;\;\;\;\;\;\;\;\;\;\;\;\;\;\;\;\;\;\;\;\;\;\;\;\;\;\;\;\;\;\;\;\;\;\;\;\;\;\;\;\;\;\;\;\;\;\;\;\;\;\;\;\;\;\;\;\;\;(500\leq T) \end{array}\right\}$$

where T is the experimental temperature, and k_E_ is the temperature-dependent reduction factor.

Yu and Kodur (2014) utilized the tangent hyperbolic (tanh) function to predict the temperature-dependent elastic modulus of CFRP reinforcement, as shown in Equation (7) [11]

(7) $$k_{E}=\frac{1+P_{R}/P_{U}}{2}-\frac{1-P_{R}/P_{U}}{2}tan\;h(k(T-T_{c}))$$

where P_R_ is the elastic modulus at the temperature at which the resin is completely decomposed (650 °C), P_U_ is the elastic modulus at room temperature, k is a constant determined through regression analysis, and T_c_ is the critical temperature at which the elastic modulus or tensile strength decreases by 50%.

In this study, the temperature at which the elastic modulus decreased to 50% of its initial value could not be clearly identified. Therefore, 375 °C was defined as the critical temperature, as this is the closest temperature at which the tensile strength dropped to approximately 50%. The measured reduction was 48% for the D10 and 58% for the D13 CFRP bars.

Zhou et al. (2019) proposed a prediction equation based on an exponential function to evaluate the variation in elastic modulus with temperature for CFRP composites, as expressed in Equation (8) [12].

(8) $$k_{E}=(1-V_{f})exp[-k_{1}(\frac{T-T_{0}}{T_{g}}{)}^{3}]+V_{f}exp[-k_{2}(\frac{T-T_{0}}{T_{d}}{)}^{3}]$$

where V_f_ is the fiber volume fraction (%), T is the temperature (°C), *T_g_* is the glass transition temperature (°C), and *T_d_* is the resin decomposition temperature (°C). The constants k_1_ and k_2_ are determined through regression analysis based on experimental data.

Ashrafi et al. (2020) proposed Equation (9) to predict the elastic modulus of GFRP composites as a function of temperature, incorporating temperature, exposure time, and composite thickness as key variables [29].

(9) $$k_{E}=-a(T{)}^{4}+b(\frac{1}{(Log(\frac{t_{1}}{6}){)}^{0.5}})-c(\frac{1}{(Log(t_{2}){)}^{0.5}})+d$$

where T is the temperature (K), t_1_ is the exposure time (min), and t_2_ is the specimen thickness (mm). The constants a, b, c, and d are determined through regression analysis based on experimental data.

**Figure 8:**
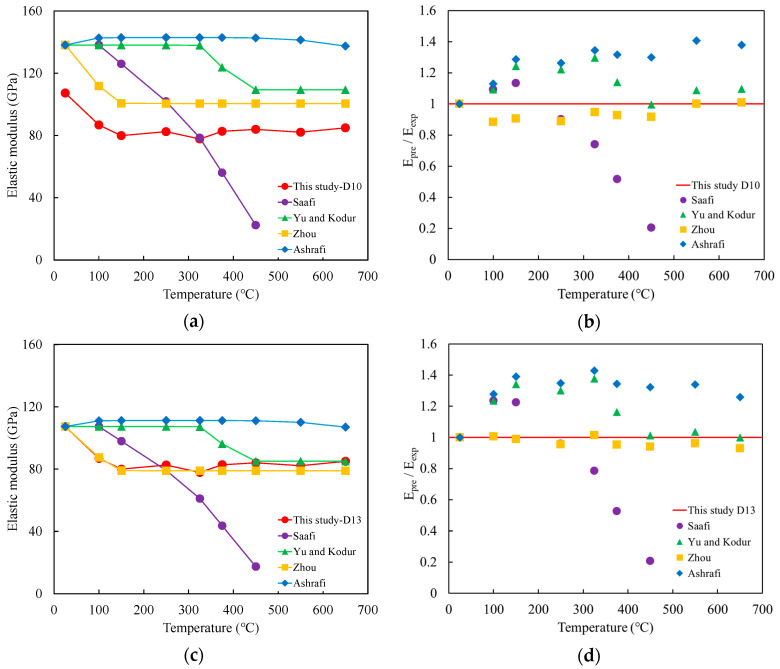
Comparison of the elastic modulus prediction models of CFRP bars. (**a**) Variation in the predicted elastic modulus for D10. (**b**) Ratio of predicted modulus to experimental modulus for D10. (**c**) Variation in the predicted elastic modulus for D13. (**d**) Ratio of predicted modulus to experimental modulus for D13.

The constants and coefficients for the prediction equation were calibrated using the experimental conditions of this study and are summarized in Table 9. The predicted elastic modulus values for the D10 and D13 CFRP bars, obtained from the proposed equation, are presented in Figure 8.

A comparative evaluation was conducted to assess the accuracy of four predictive models in estimating the elastic modulus of CFRP bars at elevated temperatures. The equation proposed by Saafi [28] yielded large discrepancies, with prediction errors ranging from approximately −100% to +24%, indicating substantial underestimation at higher temperatures. The model developed by Yu and Kodur [11] showed improved accuracy, with prediction errors between 0% and +38%, although it tended to overestimate the modulus at mid- to high-temperature ranges. In contrast, the model by Zhou et al. [12] demonstrated the closest agreement with the experimental data, with errors ranging from –7% to +1%, suggesting superior applicability under the tested conditions. The equation proposed by Ashrafi et al. [29] consistently overestimated the elastic modulus across the tested temperature range, with prediction errors ranging from +26% to +43%.

## 4. Conclusions

This study compared the elastic modulus formulas specified in ASTM D7205 and CSA S806 to analyze the elastic behavior of carbon fiber-reinforced polymer (CFRP) bars under high-temperature conditions and to evaluate their suitability for structural design applications in fire-exposed environments. The key conclusions are as follows:

1. The tensile strength and ultimate strain of CFRP bars increased up to 150 °C due to the post-curing effect. However, beyond 300 °C, both tensile strength and ultimate strain decreased sharply as a result of the thermal decomposition of the resin matrix. This degradation is attributed to the diminished stress transfer capability between the fibers and resin caused by resin decomposition at elevated temperatures.
2. In the temperature range of 150 °C to 250 °C, which lies between the glass transition temperature (127 °C) and the resin decomposition temperature (300 °C), the elastic modulus exhibited differences of up to 21.9% between values calculated using the CSA and ASTM standards. This discrepancy arises from the presence of a transition point in the stress–strain curve, where the CSA standard incorporates post-transition data, while the ASTM standard considers only the linear pre-transition segment.
3. At temperatures below 325 °C, the ASTM standard consistently defined an earlier modulus calculation interval compared to the CSA standard. Consequently, the coefficient of variation (COV) for the CSA-derived modulus values increased up to 23% due to the inclusion of post-transition data, whereas the ASTM-derived COV remained within 10%. Since the CSA standard calculates the elastic modulus based on a narrow interval (25% to 50% of the maximum load), it is less responsive to abrupt changes in the stress–strain behavior under high-temperature conditions. Therefore, further research is required to enhance the applicability of the ASTM-based CSA standard for high-temperature applications.
4. Above 375 °C, after the resin fully decomposed, the elastic modulus tended to stabilize as the load-bearing capacity became dominated by the carbon fibers. Under these conditions, the modulus values calculated using both ASTM and CSA standards showed close agreement, indicating that both standards are applicable for assessing CFRP performance at temperatures exceeding 375 °C.
5. Strength reduction factors used in design equations, as recommended by ACI 440.1R-15, were evaluated with reference to ASTM D7205 and CSA S806 standards. Both standards indicate that the strength reduction factor decreases as temperature increases, regardless of the reinforcement diameter. This reduction is primarily attributed to the thermal degradation of the epoxy resin at elevated temperatures, which compromises the bond between fibers and matrix, leading to diminished load-bearing capacity.
6. Based on the ASTM-derived elastic modulus values, prediction models proposed by the previous studies were evaluated for their predictive accuracy under high-temperature conditions. Among the evaluated models, the model incorporating material parameters such as fiber volume fraction, glass transition temperature (127 °C), and resin decomposition temperature (300 °C) showed the closest agreement with the experimental data, exhibiting the lowest average prediction error. This suggests that incorporating these parameters is effective for predicting the high-temperature modulus degradation of CFRP reinforcements.

For future work, it is suggested that further studies should investigate the detailed thermal and mechanical behavior of the epoxy matrix, CFRP rebars, and related safety factors to provide a more in-depth characterization.

## Figures and Tables

**Table 1:** Specification of CFRP rebars.

Fiber Type	Cross-Sectional Area (mm^2^)	Fiber Content (%)	Resin Type	Resin Content (%)	Glass Transition Temperature, *T_g_* (°C)	Decomposition Temperature, *T_d_* (°C)
Carbon (D10)	78.5	72.8	Epoxy	27.2	127	300
Carbon (D13)	132.7	73.5		26.5		

**Table 2:** Number of specimens.

ID	Temperature (°C)								
	25	100	150	250	325	375	450	550	650
D10 CFRP	5	5	5	5	3	3	3	3	3
D13 CFRP	5	5	5	5	3	3	3	3	3
Total	70								

**Table 3:** Test results of D10 CFRP rebar tensile test at elevated temperatures (Yun et al. [22]).

ID	Tensile Strength (MPa)	Avg. Tensile Strength (MPa)	Ultimate Strain (mm/mm)	Avg. Ultimate Strain (mm/mm)
D10−25 °C−1	2461.3	2330.8 ± 81.6	0.018	0.022 ± 0.0066
D10−25 °C−2	2336.3		0.024	
D10−25 °C−3	2240.0		0.017	
D10−25 °C−4	2367.4		0.034	
D10−25 °C−5	2249.3		0.016	
D10−100 °C−1	2381.4	2327.4 ± 39.6	0.023	0.027 ± 0.0108
D10−100 °C−2	2275.0		0.019	
D10−100 °C−3	2362.7		0.018	
D10−100 °C−4	2297.4		0.048	
D10−100 °C−5	2320.4		0.025	
D10−150 °C−1	2312.6	2292.6 ± 14.3	0.030	0.027 ± 0.0019
D10−150 °C−2	2302.5		0.026	
D10−150 °C−3	2288.1		0.025	
D10−150 °C−4	2270.4		0.025	
D10−150 °C−5	2289.3		0.026	
D10−250 °C−1	2156.1	2124.4 ± 27.1	0.019	0.023 ± 0.0017
D10−250 °C−2	2076.9		0.023	
D10−250 °C−3	2141.9		0.023	
D10−250 °C−4	2116.6		0.024	
D10−250 °C−5	2130.7		0.023	
D10−325 °C−1	2003.4	1894.6 ± 84.6	0.021	0.020 ± 0.0009
D10−325 °C−2	1797.1		0.021	
D10−325 °C−3	1883.2		0.019	
D10−375 °C−1	1230.7	1200.6 ± 33.8	0.013	0.013 ± 0.0007
D10−375 °C−2	1217.6		0.013	
D10−375 °C−3	1153.4		0.012	
D10−450 °C−1	1400.9	1258.5 ± 101.6	0.014	0.013 ± 0.0012
D10−450 °C−2	1204.3		0.012	
D10−450 °C−3	1170.3		0.012	
D10−550 °C−1	808.5	762.3 ± 51.4	0.009	0.009 ± 0.0005
D10−550 °C−2	690.7		0.008	
D10−550 °C−3	787.8		0.009	
D10−650 °C−1	414.7	426.9 ± 41.3	0.005	0.005 ± 0.0004
D10−650 °C−2	482.4		0.005	
D10−650 °C−3	383.6		0.004	

**Table 4:** Test results of D13 CFRP rebar tensile test at elevated temperatures.

ID	Tensile Strength (MPa)	Avg. Tensile Strength (MPa)	Ultimate Strain (mm/mm)	Avg. Ultimate Strain (mm/mm)
D13−25 °C−1	1690.7	1784.4 ± 134.3	0.015	0.016 ± 0.0025
D13−25 °C−2	1579.8		0.015	
D13−25 °C−3	1806.7		0.014	
D13−25 °C−4	1943.9		0.021	
D13−25 °C−5	1900.9		0.015	
D13−100 °C−1	1812.9	1839.6 ± 20.2	0.023	0.025 ± 0.0008
D13−100 °C−2	1874.7		0.025	
D13−100 °C−3	1842.4		0.025	
D13−100 °C−4	1830.7		0.024	
D13−100 °C−5	1837.4		0.025	
D13−150 °C−1	1862.5	1814.9 ± 53.4	0.037	0.029 ± 0.0055
D13−150 °C−2	1784.5		0.035	
D13−150 °C−3	1791.7		0.024	
D13−150 °C−4	1745.4		0.026	
D13−150 °C−5	1890.3		0.025	
D13−250 °C−1	1801.2	1776.3 ± 45.8	0.031	0.028 ± 0.0027
D13−250 °C−2	1719.3		0.030	
D13−250 °C−3	1765.0		0.024	
D13−250 °C−4	1850.7		0.028	
D13−250 °C−5	1745.4		0.025	
D13−325 °C−1	1404.5	1334.3 ± 54.1	0.027	0.022 ± 0.004
D13−325 °C−2	1273.0		0.019	
D13−325 °C−3	1325.4		0.020	
D13−375 °C−1	1013.4	1038.9 ± 27.1	0.015	0.015 ± 0.0004
D13−375 °C−2	1076.4		0.014	
D13−375 °C−3	1026.8		0.015	
D13−450 °C−1	1076.4	1095.9 ± 14.7	0.016	0.016 ± 0.001
D13−450 °C−2	1099.2		0.015	
D13−450 °C−3	1112.0		0.017	
D13−550 °C−1	779.9	801.0 ± 21.0	0.011	0.011 ± 0.0004
D13−550 °C−2	829.6		0.011	
D13−550 °C−3	793.3		0.010	
D13−650 °C−1	319.8	324.3 ± 18.5	0.005	0.005 ± 0.0004
D13−650 °C−2	304.2		0.005	
D13−650 °C−3	348.8		0.005	

**Table 5:** Elastic modulus of D10 CFRP rebars at elevated temperatures according to codes.

ID	CSA S806				ASTM D7205				CSA/ASTM
	Elastic Modulus (GPa)	Avg. (GPa)	SD (GPa)	COV (%)	Elastic Modulus (GPa)	Avg. (GPa)	SD (GPa)	COV (%)	Error (%)
D10−25 °C−1	134.6	143.4	5.7	4.0	137.8	138.1	2.1	1.6	103.8
D10−25 °C−2	147.2				141.6				
D10−25 °C−3	149.8				139.0				
D10−25 °C−4	146.4				135.1				
D10−25 °C−5	139.0				137.3				
D10−100 °C−1	127.9	115.7	13.4	11.5	137.6	126.3	11.3	8.9	91.6
D10−100 °C−2	121.3				127.7				
D10−100 °C−3	129.8				138.9				
D10−100 °C−4	98.9				109.8				
D10−100 °C−5	100.5				117.7				
D10−150 °C−1	98.3	98.7	1.3	1.3	111.8	111.1	1.8	1.7	88.8
D10−150 °C−2	98.8				111.8				
D10−150 °C−3	101.0				113.9				
D10−150 °C−4	98.1				109.4				
D10−150 °C−5	97.2				108.8				
D10−250 °C−1	117.2	101.9	8.2	8.1	131.8	113.2	10.5	9.2	90.0
D10−250 °C−2	100.3				114.2				
D10−250 °C−3	100.9				109.7				
D10−250 °C−4	92.5				99.9				
D10−250 °C−5	98.4				110.2				
D10−325 °C−1	100.5	98.9	4.0	4.0	110.0	106.3	4.9	4.6	93.0
D10−325 °C−2	93.5				99.4				
D10−325 °C−3	102.8				109.5				
D10−375 °C−1	108.5	106.6	3.0	2.8	111.1	108.6	3.1	2.8	98.2
D10−375 °C−2	102.3				104.2				
D10−375 °C−3	109.0				110.3				
D10−450 °C−1	107.4	107.6	0.2	0.2	110.5	109.8	0.5	0.4	98.0
D10−450 °C−2	107.6				109.3				
D10−450 °C−3	107.9				109.7				
D10−550 °C−1	95.3	99.8	3.2	3.2	96.3	100.5	3.0	3.0	99.3
D10−550 °C−2	102.2				102.3				
D10−550 °C−3	101.9				102.9				
D10−650 °C−1	99.7	99.8	0.9	0.9	99.7	99.7	0.5	0.5	100.1
D10−650 °C−2	98.8				99.1				
D10−650 °C−3	101.0				100.2				

**Table 6:** Elastic modulus of D13 CFRP rebars at elevated temperatures according to codes.

ID	CSA S806				ASTM D7205				CSA/ASTM
	Elastic Modulus (GPa)	Avg. (GPa)	SD (GPa)	COV (%)	Elastic Modulus (GPa)	Avg. (GPa)	SD (GPa)	COV (%)	Error (%)
D13−25 °C−1	111.6	105.2	19.0	18.0	108.3	107.3	10.6	9.9	98.0
D13−25 °C−2	97.6				96.6				
D13−25 °C−3	127.3				121.2				
D13−25 °C−4	72.5				94.1				
D13−25 °C−5	117.1				116.3				
D13−100 °C−1	79.2	77.2	2.1	2.7	89.1	86.8	3.1	3.5	88.9
D13−100 °C−2	79.2				90.3				
D13−100 °C−3	75.2				83.8				
D13−100 °C−4	78.1				88.3				
D13−100 °C−5	74.3				82.5				
D13−150 °C−1	46.11	62.0	10.6	17.1	83.0	80.8	6.1	7.6	76.7
D13−150 °C−2	53.05				69.1				
D13−150 °C−3	74.13				86.9				
D13−150 °C−4	69.25				82.0				
D13−150 °C−5	67.62				83.2				
D13−250 °C−1	58.8	65.5	11.1	16.9	79.3	82.5	2.7	3.3	79.4
D13−250 °C−2	49.7				82.7				
D13−250 °C−3	75.7				82.8				
D13−250 °C−4	63.3				80.6				
D13−250 °C−5	79.9				87.2				
D13−325 °C−1	46.02	68.1	15.7	23.0	69.1	77.8	6.4	8.3	87.5
D13−325 °C−2	77.52				79.8				
D13−325 °C−3	80.69				84.4				
D13−375 °C−1	79.3	81.3	1.4	1.7	81.3	82.7	1.4	1.7	98.3
D13−375 °C−2	82.9				84.7				
D13−375 °C−3	80.7				82.2				
D13−450 °C−1	83.93	82.0	1.5	1.9	85.4	83.9	1.0	1.2	97.74
D13−450 °C−2	80.15				83.5				
D13−450 °C−3	81.80				82.9				
D13−550 °C−1	80.89	81.1	2.3	2.8	81.7	82.1	1.8	2.1	98.8
D13−550 °C−2	78.38				80.2				
D13−550 °C−3	83.90				84.4				
D13−650 °C−1	83.40	84.9	1.2	1.4	83.2	84.9	1.3	1.5	100
D13−650 °C−2	86.33				86.0				
D13−650 °C−3	84.96				85.7				

**Table 7:** Guaranteed tensile strength and elastic modulus of CFRP rebars based on CSA S806.

ID	CSA S806			
	Guaranteed Tensile Strength (MPa)	GTS */ATS **	Guaranteed Elastic Modulus (MPa)	GEM ***/AEM ****
D10−25 °C	2141.42	0.92	143.4	1.00
D10−100 °C	2234.29	0.96	86.3	0.75
D10−150 °C	2258.71	0.99	98.7	1.00
D10−250 °C	2060.48	0.97	83.5	0.82
D10−325 °C	1684.02	0.89	98.9	1.00
D10−375 °C	1115.18	0.93	106.6	1.00
D10−450 °C	1013.65	0.81	107.6	1.00
D10−550 °C	636.96	0.84	99.8	1.00
D10−650 °C	328.75	0.77	99.8	1.00
D13−25 °C	1481.45	0.83	65.2	0.62
D13−100 °C	1791.90	0.97	77.2	1.00
D13−150 °C	1690.47	0.93	39.5	0.64
D13−250 °C	1669.30	0.94	42.0	0.64
D13−325 °C	1198.41	0.90	34.6	0.51
D13−375 °C	970.28	0.93	81.3	1.00
D13−450 °C	1058.24	0.97	82.0	1.00
D13−550 °C	747.83	0.93	81.1	1.00
D13−650 °C	278.76	0.86	84.9	1.00

GTS *: guaranteed tensile strength; ATS **: average tensile strength; GEM ***: guaranteed elastic modulus; AEM ****: average elastic modulus.

**Table 8:** Guaranteed tensile strength and elastic modulus of CFRP rebars based on ASTM D7205.

ID	ASTM D7205			
	Guaranteed Tensile Strength (MPa)	GTS */ATS **	Guaranteed Elastic Modulus (MPa)	GEM ***/AEM ****
D10−25 °C	2086	0.89	138.1	1.00
D10−100 °C	2208.6	0.95	126.3	1.00
D10−150 °C	2249.7	0.98	111.1	1.00
D10−250 °C	2043.1	0.96	113.2	1.00
D10−325 °C	1640.8	0.87	106.3	1.00
D10−375 °C	1099.2	0.92	108.6	1.00
D10−450 °C	953.7	0.76	109.8	1.00
D10−550 °C	608.1	0.80	100.5	1.00
D10−650 °C	303	0.71	99.7	1.00
D13−25 °C	1381.5	0.77	107.3	1.00
D13−100 °C	1779	0.97	86.8	1.00
D13−150 °C	1654.7	0.91	80.8	1.00
D13−250 °C	1638.9	0.92	82.5	1.00
D13−325 °C	1171.1	0.88	77.8	1.00
D13−375 °C	957.6	0.92	82.7	1.00
D13−450 °C	1051.8	0.96	83.9	1.00
D13−550 °C	738	0.92	82.1	1.00
D13−650 °C	268.8	0.83	84.9	1.00

GTS *: guaranteed tensile strength; ATS **: average tensile strength; GEM ***: guaranteed elastic modulus; AEM ****: average elastic modulus.

**Table 9:** Elastic modulus prediction equation at elevated temperatures.

Previous Study	Prediction Model
Saafi [28]	$k_{E}=\left\{\begin{array}{c} 1\;\;\;\;\;\;\;\;\;\;\;\;\;\;\;\;\;\;\;\;\;\;\;\;\;\;\;\;\;\;\;\;\;\;\;\;\;\;\;\;\;\;\;\;\;\;\;\;\;\;\;\;\;\;\;\;\;\;\;\;\;\;\;(0\leq T\leq 100\;{}^{\circ}C)\\ 1.175-0.00175T\;\;\;\;\;\;\;\;\;\;\;\;\;\;\;\;\;\;\;\;\;\;\;\;\;\;\;(100\;{}^{\circ}C\leq T\leq 300\;{}^{\circ}C)\\ 1.625-0.00325T\;\;\;\;\;\;\;\;\;\;\;\;\;\;\;\;\;\;\;\;\;\;\;\;\;\;\;(300\;{}^{\circ}C\leq T\leq 500\;{}^{\circ}C)\\ 0\;\;\;\;\;\;\;\;\;\;\;\;\;\;\;\;\;\;\;\;\;\;\;\;\;\;\;\;\;\;\;\;\;\;\;\;\;\;\;\;\;\;\;\;\;\;\;\;\;\;\;\;\;\;\;\;\;\;\;\;\;\;\;\;\;\;\;\;\;\;\;\;\;\;\;\;\;(500\leq T) \end{array}\right\}$
Yu and Kodur [11]	$k_{E}=0.896-0.104tan\;h(0.0514(T-375))$
Zhou et al. [12]	$D10\;\;\;\;\;\;k_{E}=(1-0.728)exp[-5.86(\frac{T-25}{127}{)}^{3}]+0.728exp[-1\times 10^{-18}(\frac{T-25}{300}{)}^{3}]$ $D13\;\;\;\;\;\;k_{E}=(1-0.735)exp[-5.86(\frac{T-25}{127}{)}^{3}]+0.735exp[-1\times 10^{-18}(\frac{T-25}{300}{)}^{3}]$
Ashrafi et al. [29]	$k_{E}=-1.9644\times 10^{-12}(T-273{)}^{4}+0.02847(\frac{1}{(Log(\frac{t_{1}}{6}){)}^{0.5}})-0.024488(\frac{1}{(Log(t_{2}){)}^{0.5}})+1.02523$

## Data Availability

The original contributions presented in the study are included in the article; further inquiries can be directed to the corresponding author.
